# Universal nanohydrophobicity predictions using virtual nanoparticle library

**DOI:** 10.1186/s13321-019-0329-8

**Published:** 2019-01-18

**Authors:** Wenyi Wang, Xiliang Yan, Linlin Zhao, Daniel P. Russo, Shenqing Wang, Yin Liu, Alexander Sedykh, Xiaoli Zhao, Bing Yan, Hao Zhu

**Affiliations:** 1 The Rutgers Center for Computational and Integrative Biology, Camden, NJ 08102 USA; 2 School of Chemistry and Chemical Engineering, Shandong University, Jinan, 250100 China; 3 Research Center for Eco-Environmental Science, Chinese Academy of Sciences, Beijing, 100085 China; 4 Sciome, Research Triangle Park, NC 27709 USA; 5 Department of Physiological Sciences, Eastern Virginia Medical School, Norfolk, VA 23507 USA; 6 School of Environment, Jinan University, Guangzhou, 510632 China; 7 Department of Chemistry, Rutgers University, 315 Penn St., Camden, NJ 08102 USA; 8 College of Life Science and Bio-Engineering, Beijing University of Technology, Beijing, 100124 China

**Keywords:** Nanohydrohobicity, Surface chemistry, Surface simulations, Nanomaterials design, Virtual nanoparticle library, Predictive model

## Abstract

To facilitate the development of new nanomaterials, especially nanomedicines, a novel computational approach was developed to precisely predict the hydrophobicity of gold nanoparticles (GNPs). The core of this study was to develop a large virtual gold nanoparticle (vGNP) library with computational nanostructure simulations. Based on the vGNP library, a nanohydrophobicity model was developed and then validated against externally synthesized and tested GNPs. This approach and resulted model is an efficient and effective universal tool to visualize and predict critical physicochemical properties of new nanomaterials before synthesis, guiding nanomaterial design.

**Electronic supplementary material:**

The online version of this article (10.1186/s13321-019-0329-8) contains supplementary material, which is available to authorized users.

Advances in nanotechnology and material sciences in the past decade have led to the rapid development of engineered nanomedicines in pharmaceutical sciences [1, 2]. The traditional development route of new nanomaterials solely depends on experimental testing, which is costly and time consuming. With rapidly rising experimental and labor costs, computational approaches have become promising low cost alternatives to study nanomaterials [3]. To date, computational modeling approaches are broadly applied to the research and development procedure of small molecules, but rarely for larger molecules like nanomaterials [4]. This is evidenced by the many available commercial software tools [5–7] capable of predicting physicochemical properties for new druggable small molecules but none are available for new nanomedicines. Compared to small molecules, the shape, size, composition and surface ligands of nanomaterials greatly increase nanostructure complexity. Due to this increased complexity, the biological activities and therapeutic effects of nanomaterials are more difficult to model than small molecules. As a key determinant of drug pharmacokinetics, hydrophobicity influences drug solubility, absorption, distribution, and target binding characteristics, which are eventually associated with the drug efficacy, potency and toxicity [8, 9]. Therefore, it is critical to evaluate the hydrophobicity of nanomedicines in the early stages of development, even before chemical synthesis.

In previous studies, researchers have been devoted to building quantitative structure activity relationship (QSAR) models for various bioactivities of different nanomaterials but have had limited applicability for new nanomaterial development [10–13]. Namely, two major issues limited the applicability of the resulted models: (1) the lack of enough high quality nano-bioactivity data and (2) computational approaches to precisely quantify nanostructure diversity. Currently, the use of experimental values as descriptors [14, 15] prevents the predictions of new nanomaterials before chemical synthesis. On the other hand, computational calculation of descriptors allows for virtual nanoparticle generation and nano-bioactivity prediction with no chemical synthesis required. Some researchers found that descriptors calculated solely from the surface ligands of nanoparticles were useful in predicting properties. Although this is useful in predicting certain properties of nanoparticles, the effects of the nanoparticle size and surface ligands density, position, distribution, were not considered in these studies and likely also contribute to the nano-bioactivity. More recently, however, some researchers have utilized some of these properties in addition to the general descriptor set from surface ligands, e.g., electronic properties, [16] ionic characteristics, [17] and others [18–20]. The major drawback of these available modeling studies is the lack of approaches to correctly quantify and represent nanostructure diversity during the modeling procedure. In our previous studies, we have shown that surface chemistry was the most critical factor in determining the bioactivities of gold nanoparticles (GNPs), including nanohydrophobicity [21]. Furthermore, correctly simulating surface chemistry can result in novel nanodescriptors which can be used to develop quantitative nanostructure–activity relationship (QNAR) models, showing superior advantages than traditional modeling studies [22]. Here, we report a novel approach to develop a virtual gold nanoparticle (vGNP) library with surface simulations precisely predicting nanohydrophobicity for new nanomaterials. Using this approach, a nanohydrophobicity model was developed based on surface chemistry simulation of a set of GNPs with various surface ligands. The model predictivity was further proved by experimentally synthesizing and testing nine new GNPs, and comparing their experimental/predicted logP values. The predicted nanohydrophobicity showed high correlations with experimental results, indicating the applicability of using this universal predictive modeling approach to design and select new GNPs with desired hydrophobicity.

In a recent study, we developed a novel method to construct vGNP libraries [22]. Using this approach, we constructed the vGNP library with a dataset of 41 GNPs, as shown in Fig. 1. Specifically, using the structural information of surface ligands, ligand density of each GNP, and the GNP size, the virtual structure for each of the GNPs in the library was constructed as follows. First, the gold core was constructed based on the GNP size. Then, the surface ligands, with ligand density information, were randomly attached to the gold core to simulate the experimental conditions. These 41 GNPs were synthesized and tested for their hydrophobicity. The high nanostructure diversity of these 41 GNPs, including various surface ligands, different ligand densities per GNP and various GNP sizes, and high hydrophobicity diversity (experimental logP values range from − 3 to 3) make this dataset suitable for modeling purposes. This dataset was used as the modeling set to develop nanohydrophobicity models. The experimental approaches to synthesize this GNP library and test the logP values are described in our previous study [22]. All the experimental data used to construct the vGNP library, including the structure information of surface ligands, are provided in Additional file 4: Table SI.

**Fig. 1:**
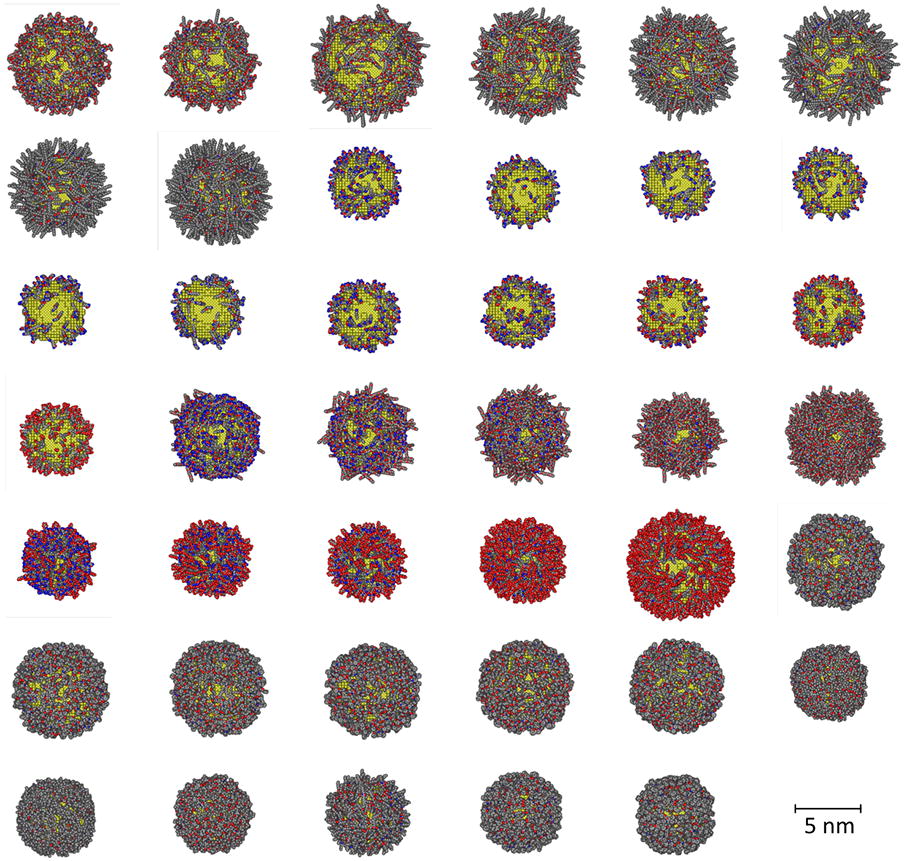
The constructed vGNP library

Besides providing a large nanohydrophobicity dataset in this study, a new surface chemistry simulation approach was developed based on the constructed vGNP library to evaluate hydrophobicity of GNPs. The core of this technique was to evaluate the solvent accessible surface (SAS) of GNPs and to calculate the nanohydrophobicity accordingly. The SAS, also named the Connolly Surface, [23] was identified for each GNP using a grid based method [24]. The cross section (grey area) of a vGNP surface ligand was constructed in a 2D grid as shown in Fig. 2a. The SAS was determined by rolling a solvent probe, simulated by the size of a water molecule of radius 1.4 Å, over the surface of the vGNP. Probes were placed on grid points surrounding the vGNP surface ligand. A grid point was identified as a SAS point of this vGNP when the probe was within one grid unit distance to at least one vGNP atom, and does not overlap with any other vGNP atoms [24].

**Fig. 2:**
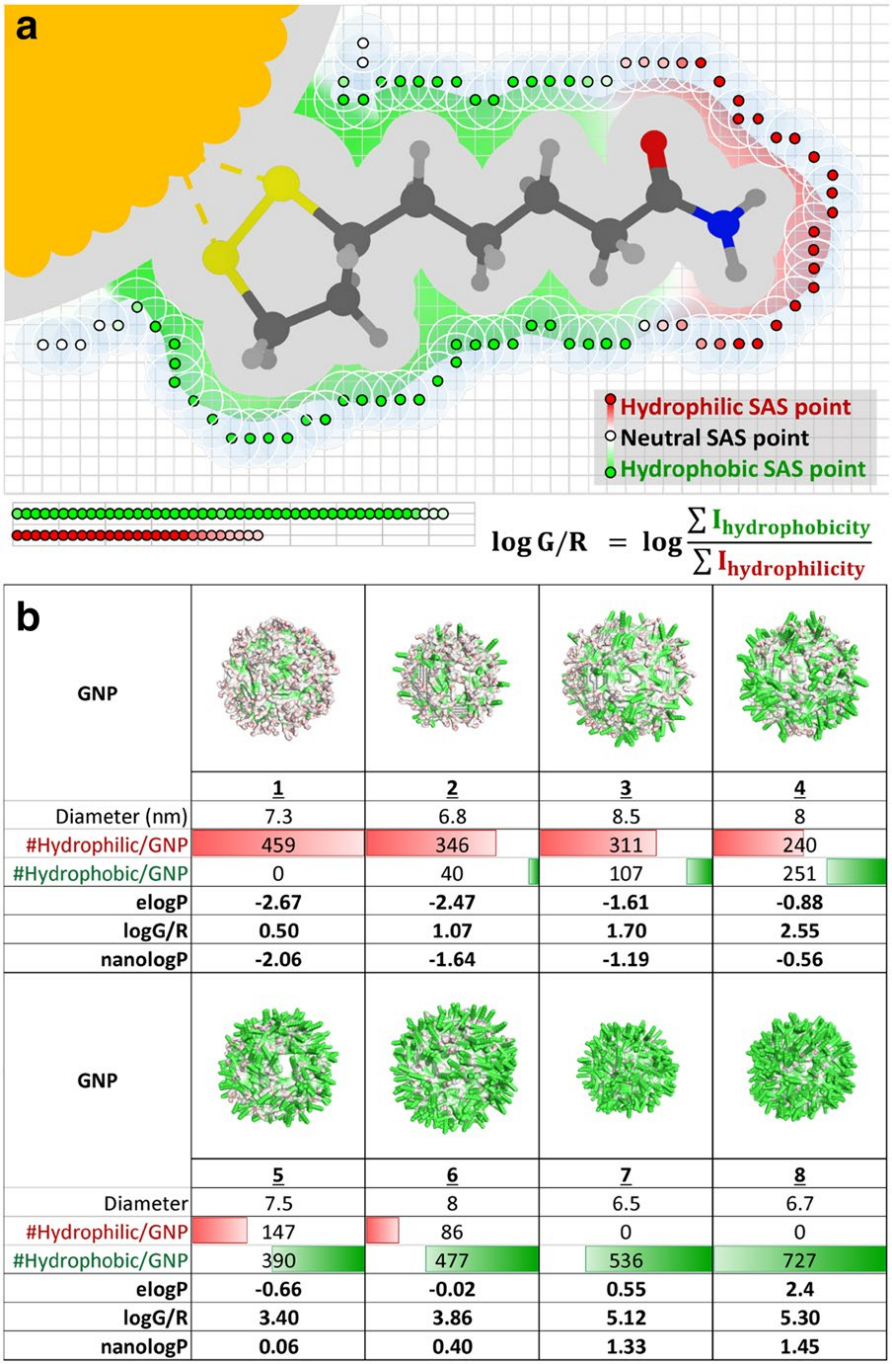
Illustration of nanologP evaluations. **a** The SAS surface identified by rolling the solvent probe on the vGNP surface, and hydrophobicity potentials represented as colors. **b** A series of vGNPs with various calculated nanologP values

Once the SAS, with all identified grid points, was constructed for a vGNP, its hydrophobicity potential was evaluated by calculating the octanol–water partition coefficient from a distance-dependent weighting function of atomic contributions [25, 26]. The hydrophobic/hydrophilic potential of an identified SAS point was determined by nearby atoms and weighted by their distances to the SAS point. As shown in Fig. 2a, hydrophilic SAS points were colored with red while hydrophobic SAS points were colored with green. The hydrophilic/hydrophobic potential for each SAS was represented as the intensity of the corresponding color—red as hydrophilic and green as hydrophobic. As an example, the hydrophobic potentials of eight vGNPs can be visualized in Fig. 2b. This series of GNPs were constructed with two types of surface ligands with different hydrophobicities: one ligand was hydrophilic and the other was hydrophobic. The ratio of these two types of surface ligands among the eight GNPs was gradually changed to modulate the nanohydrophobicity from low to high. From Fig. 2b, this series of GNPs showed a clear trend of hydrophobicity change with an increased ratio of hydrophilicity/hydrophobicity surface ligands. Thus, the surface colored vGNPs could be a representation of nanohydrophobicity of GNPs.

The nanohydrophobicity was then quantified using the colored vGNP. The nanohydrophobicity of a vGNP can be calculated as:

1 $$\log G /R = \log \frac{{\sum I_{\text{hydrophobicity}} }}{{\sum I_{\text{hydrophilicity}} }}$$

where G and R represent the hydrophobic potential (green) and hydrophilic potential (red) for each SAS point, and I is the intensity of hydrophobic/hydrophilic potential.

Then, with a linear regression analysis between logG/R and logP values of these 41 GNPs, the following equation was generated and can be used to calculate nanologP (i.e. logP values of GNPs) values for new GNPs from their logG/R results, which were obtained from vGNP simulations:

2 $${\text{nanolog}}P = 0.7334*\log G /R - 2.4306$$

The calculated logP values of all the 41 nanoparticles (nanologP), obtained from the above equation, were compared to their experimental logP results (elogP), which were obtained by experimentally testing the partition coefficients in n-octanol and water solutions.

The step by step instruction of vGNP generations and log G/R calculations were described in the Additional file 3 (vGNP logP Supplementary demo file) and all source code files were also shared as Python files (see details in the Additional files 1, 2, and 3).

In some previous studies, logP of nanomaterials were calculated based only on surface ligand structures [15, 18, 19, 21, 27]. For comparison purposes, logP values of these 41 GNPs were calculated using four calculators, XlogP3, [28] AlogPS 2.1, [29] ClogP calculated in ChemDraw 17.0 [30] and the logP model in MOE 2016 [31]. These four logP calculators were built by either chemical atom/fragment contribution methods (XlogP3, ClogP and logP in MOE) or QSAR modeling (AlogPS). These calculators are commonly used to calculate the surface ligand logP and are based on various linear and non-linear modeling approaches. For example, XlogP3, AlogPS 2.1, ClogP and logP in MOE were based on a nearest neighbor approach combined with linear additive model [28], associated neural networks [29], fragmental additive approach [30] and atom additive approach [31], respectively. When modeling mixtures, the weighted average according to the component fractions was used for calculating the chemical descriptors [32]. Similarly, in this study, for a GNP with two different surface ligands, its logP value was calculated by averaging two ligand logP values weighted by the number of the two types of ligands. As shown in Fig. 3 and Additional file 4: Table SI, the best obtained logP results from commercial software, XlogP3, which yielded a low correlation with elogP with a coefficient of determination (R^2^) = 0.577, and large prediction errors as Mean Absolute Error (MAE) = 2.633 and root mean square error (RMSE) = 3.00 [33, 34]. These results were much worse than that of nanologP developed in this study (R^2^ = 0.884, MAE = 0.719 and RMSE = 0.81). A five-fold cross-validation was performed for nanologP and the results are similar (R^2^ = 0.832, MAE = 0.75 and RMSE = 1.28). The summary table of training and validation sets and the orginal GNP library file for calculation can be viewed in Additional files 4 and 5.

**Fig. 3:**
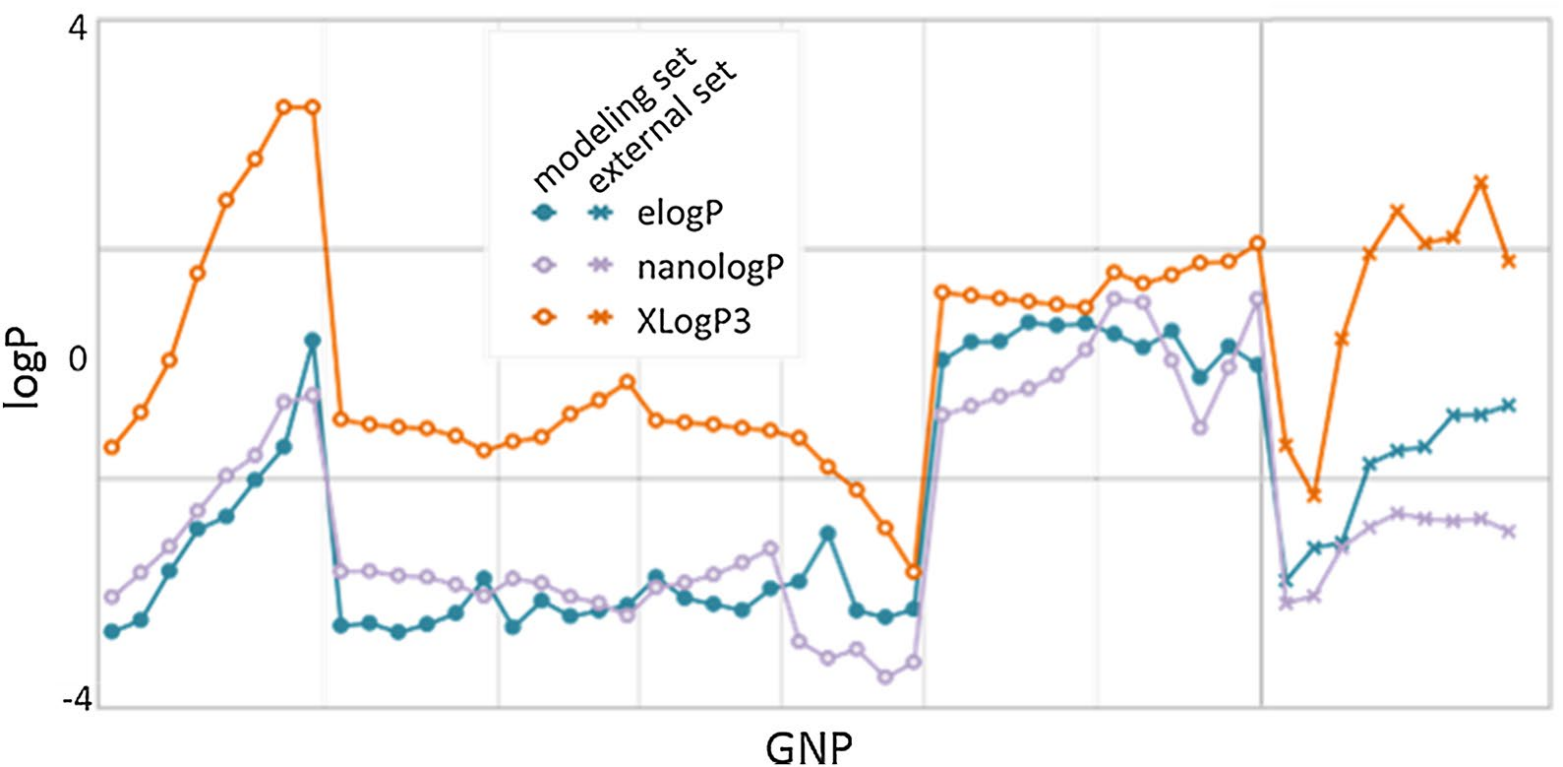
Comparing the accuracy of calculated nanologP and commercial XLogP3

To further validate the performance of the proposed nanologP method, we synthesized nine new GNPs with different surface ligands compared to the modeling set and experimentally obtained their elogP values. The calculated nanologP values show high predictivity for this external set with R_ext_^2^ = 0.762, MAE_ext_ = 1.182 and RMSE_ext_ = 1.24, similar to the modeling set result. In comparison, the best calculated logP values from commercial software (XlogP3) show much worse prediction accuracy with R_ext_^2^ = 0.534, MAE_ext_ = 3.097 and RMSE_ext_ = 3.49.

In this study, an applicable nanohydrophobicity computational method was developed. The results showed that precisely simulated nanostructures using the vGNP library technique was the key to the accurate calculation of physicochemical properties of GNPs, such as hydrophobicity. There is potential to adapt the approach for other nanoparticles (e.g., carbon nanotubes and silver nanoparticles). The logG/R can be calculated by simulating the new type of nanoparticles with the designated core and shape, and the same hydrophobicity/hydrophilicity evaluation strategy. This is an ongoing work when more experimental data becomes available in the future. Furthermore, this approach can also be applied to the modeling and evaluation of other critical properties or bioactivities (e.g., interaction potentials with the environment, permeability through cell membranes, etc.).

## Additional files

**Additional file 1.** Python codes for the calculation of nanologP.

**Additional file 2.** Python codes for the calculation of nanologP.

**Additional file 3.** A demo for calculation of nanologP.

**Additional file 4.** Summary table of the GNP library in the training and validation sets.

**Additional file 5.** The original GNP library input file for the demo.

## Abbreviations

GNP: gold nanoparticle
vGNP: virtual gold nanoparticle
QSAR: quantitative structure activity relationship
QNAR: quantitative nanostructure-activity relationship
SAS: solvent accessible surface

## References

[1] Shi J, Votruba AR, Farokhzad OC, Langer R (2010). Nanotechnology in drug delivery and tissue engineering: from discovery to applications. Nano Lett.

[2] Zhang L, Gu FX, Chan JM (2008). Nanoparticles in medicine: therapeutic applications and developments. Clin Pharmacol Ther.

[3] Winkler DA, Mombelli E, Pietroiusti A (2013). Applying quantitative structure–activity relationship approaches to nanotoxicology: current status and future potential. Toxicology.

[4] Oberdörster G (2010). Safety assessment for nanotechnology and nanomedicine: concepts of nanotoxicology. J Intern Med.

[5] Pires DEV, Blundell TL, Ascher DB (2015). pkCSM: predicting small-molecule pharmacokinetic and toxicity properties using graph-based signatures. J Med Chem.

[6] Hansen K, Biegler F, Ramakrishnan R (2015). Machine learning predictions of molecular properties: accurate many-body potentials and nonlocality in chemical space. J Phys Chem Lett.

[7] Lagorce D, Sperandio O, Baell JB (2015). FAF-Drugs3: a web server for compound property calculation and chemical library design. Nucleic Acids Res.

[8] Krämer SD, Wunderli-Allenspach H (2001). Physicochemical properties in pharmacokinetic lead optimization. Farmaco.

[9] Gleeson MP (2008). Generation of a set of simple, interpretable ADMET rules of thumb. J Med Chem.

[10] Walkey CD, Olsen JB, Song F (2014). Protein corona fingerprinting predicts the cellular interaction of gold and silver nanoparticles. ACS Nano.

[11] Chen R, Zhang Y, Monteiro-Riviere NA, Riviere JE (2016). Quantification of nanoparticle pesticide adsorption: computational approaches based on experimental data. Nanotoxicology.

[12] Pathakoti K, Huang MJ, Watts JD (2014). Using experimental data of Escherichia coli to develop a QSAR model for predicting the photo-induced cytotoxicity of metal oxide nanoparticles. J Photochem Photobiol B Biol.

[13] Fourches D, Pu D, Tassa C (2010). Quantitative nanostructure − activity relationship modeling. ACS Nano.

[14] Mikolajczyk A, Malankowska A, Nowaczyk G (2016). Combined experimental and computational approach to developing efficient photocatalysts based on Au/Pd–TiO_2_ nanoparticles. Environ Sci Nano.

[15] Fourches D, Pu D, Li L (2016). Computer-aided design of carbon nanotubes with the desired bioactivity and safety profiles. Nanotoxicology.

[16] Jagiello K, Chomicz B, Avramopoulos A (2017). Size-dependent electronic properties of nanomaterials: How this novel class of nanodescriptors supposed to be calculated?. Struct Chem.

[17] Sizochenko N, Mikolajczyk A, Jagiello K (2018). How the toxicity of nanomaterials towards different species could be simultaneously evaluated: a novel multi-nano-read-across approach. Nanoscale.

[18] Toropov AA, Toropova AP, Puzyn T (2013). QSAR as a random event: modeling of nanoparticles uptake in PaCa2 cancer cells. Chemosphere.

[19] Luan F, Tang L, Zhang L (2016). A further development of the QNAR model to predict the cellular uptake of nanoparticles by pancreatic cancer cells. Food Chem Toxicol.

[20] Mikolajczyk A, Pinto HP, Gajewicz A (2015). Ab initio studies of anatase TiO_2_ (101) surface-supported Au8 clusters. Curr Top Med Chem.

[21] Li S, Zhai S, Liu Y (2015). Experimental modulation and computational model of nano-hydrophobicity. Biomaterials.

[22] Wang W, Sedykh A, Sun H (2017). Predicting nano-bio interactions by integrating nanoparticle libraries and quantitative nanostructure activity relationship modeling. ACS Nano.

[23] Connolly M (1983). Solvent-accessible surfaces of proteins and nucleic acids. Science.

[24] Sethian JA (1998). Fast marching methods and level set methods for propagating interfaces. Proc Natl Acad Sci USA.

[25] Wildman SA, Crippen GM (1999). Prediction of physicochemical parameters by atomic contributions. J Chem Inf Comput Sci.

[26] Heiden W, Moeckel G, Brickmann J (1993). A new approach to analysis and display of local lipophilicity/hydrophilicity mapped on molecular surfaces. J Comput Aided Mol Des.

[27] Moyano DF, Goldsmith M, Solfiell DJ (2012). Nanoparticle hydrophobicity dictates immune response. J Am Chem Soc.

[28] Cheng T, Zhao Y, Li X (2007). Computation of octanol–water partition coefficients by guiding an additive model with knowledge. J Chem Inf Model.

[29] Tetko IV, Tanchuk VY (2002). Application of associative neural networks for prediction of lipophilicity in ALOGPS 2.1 program. J Chem Inf Comput Sci.

[30] BioByte. http://www.biobyte.com/. Accessed 22 Feb 2018

[31] Chemical Computing Group ULC (2013) Molecular operating environment (MOE)

[32] Muratov EN, Varlamova EV, Artemenko AG (2012). Existing and developing approaches for QSAR analysis of mixtures. Mol Inform.

[33] Sizochenko N, Jagiello K, Leszczynski J, Puzyn T (2015). How the “liquid drop” approach could be efficiently applied for quantitative structure-property relationship modeling of nanofluids. J Phys Chem C.

[34] Mikolajczyk A, Sizochenko N, Mulkiewicz E (2017). Evaluating the toxicity of TiO_2_-based nanoparticles to Chinese hamster ovary cells and *Escherichia coli*: a complementary experimental and computational approach. Beilstein J Nanotechnol.

