# Supplementary material for: Universal nanohydrophobicity predictions using virtual nanoparticle library
Source: J Cheminform. 2019 Jan 18;11:6. doi: 10.1186/s13321-019-0329-8 (PMC6689884; doi:10.1186/s13321-019-0329-8)
Supplement: Supplementary file 3 — Additional file 3. A demo for calculation of nanologP. [file 13321_2019_329_MOESM3_ESM.docx]

**Universal Nanohydrophobicity Predictions using Virtual Nanoparticle Library**

Wenyi Wang^1^, Xiliang Yan^1,2^, Linlin Zhao^1^, Daniel P. Russo^1^, Shenqing Wang^2^, Yin Liu^3^, Alexander Sedykh^1,4^, Xiaoli Zhao^5^, Bing Yan^2,6^ and Hao Zhu ^1,7^*

^1^ The Rutgers Center for Computational and Integrative Biology, Camden, New Jersey 08102, USA;

^2^ School of Chemistry and Chemical Engineering, Shandong University, Jinan, 250100, China;

^3^ Research Center for Eco-Environmental Science, Chinese Academy of Sciences, Beijing 100085, China;

^4^ Sciome, Research Triangle Park, NC 27709, USA;

^5^ Department of Physiological Sciences, Eastern Virginia Medical School, Norfolk, Virginia 23507, USA;

^6^ School of Environment, Jinan University, Guangzhou 510632, China

^7^ Department of Chemistry, Rutgers University, Camden, New Jersey 08102, USA;

*Corresponding authors:

Hao Zhu

315 Penn St.

Rutgers University

Camden, NJ 08102

Telephone: (856) 225-6781

Email: hao.zhu99@rutgers.edu

**How to create vGNP library and calculate logGR**

**1. Run the GNPrep to generate the vGNPs in pdb format.**

The input sdf file, univ_multi.sdf, where each ligand includes the following data fields: CAS, the index of the GNP, graph.index, the number of ligands on each GNP, and dipole, the nanoparticle radius in Angstrom. The consecutive molecules with same CAS are the multiple types of surface ligand on one vGNP. Open a command line window in the same directory and type python gnprep.py univ_multi.sdf. Output are individual pdb file, one GNP per file.

**2. Create lipophilicity surface**

Open each pdb file in MOE. Go to Surface - surfaces and maps. In the pop-up window, select atoms: all atoms, near: all atoms, color: lipophilicity, Hydrophilic: (click the double down arrow for more options, red: 255, green: 0, blue: 0) pure red, Lipophilic: (click the double down arrow for more options, red: 0, green: 255, blue: 0) pure green. Then click create. When the surface is created, go to the menu: File - Save - file name as: 1_lipophilicity.moe 2_lipophilicity.moe etc.

**3. Create logGR descriptor**

Create descriptor file. First prepare a list.txt file to be placed in the same folder. The file should include three columns: CAS, dipole, and elogP. Finally, run the python code: colorQuantification.py, descriptors are in the output logGR_Ratio.txt file, third column and are compared to the experimental logP (second column)
